# Parent-Implemented Hanen Program It Takes Two to Talk^®^: An Exploratory Study in Spain

**DOI:** 10.3390/ijerph18158214

**Published:** 2021-08-03

**Authors:** Nuria Senent-Capuz, Inmaculada Baixauli-Fortea, Carmen Moret-Tatay

**Affiliations:** 1 Department of Occupational Sciences, Speech Language Pathology and Developmental and Educational Psychology, Faculty of Psychology, Catholic University of Valencia San Vicente Mártir (UCV), Av. de la Ilustración, 2, 46100 Burjassot, Spain; inmaculada.baixauli@ucv.es; 2 Faculty of Psychology International Coordinator, Psychology, Catholic University of Valencia San Vicente Mártir, Av. de la Ilustración, 2, 46100 Burjassot, Spain; mariacarmen.moret@ucv.es; 3 Dipartimento di Neuroscienze Salute Mentale e Organi di Senso (NESMOS), La Sapienza University of Rome, 00185 Rome, Italy

**Keywords:** parent-implemented intervention, expressive language delay, late talkers, early language intervention, Hanen program, parent stress, parent perceptions

## Abstract

Parent-implemented interventions are a highly common approach for enhancing communication and linguistic abilities of late talkers, involving a population that shows a small expressive vocabulary in the absence of other deficits that could explain it. This study aimed to compare the outcomes of a parent-implemented language intervention, It Takes Two to Talk^®^—The Hanen Program^®^ for Parents (ITTT), to a clinician-directed therapy. Participants were 17 families and their late-talking children: 10 families took part in ITTT and 7 in the clinician-directed modality. The outcomes in the social communication domain were more favorable for the ITTT group, but there were no significant differences between groups as regards vocabulary and syntax. In terms of parents, the research focused on examining if there were significant changes in parents’ stress and their perceptions of their children’s communication abilities. No differences were observed in the level of stress. In contrast, the group that received the ITTT program significantly altered their perceptions of their children’s communication difficulties in comparison with the clinician-directed therapy. These results have implications in the clinical management of late-talking children, and they are discussed in terms of evidence-based practice.

## 1. Introduction

Parent-implemented interventions have become an essential component of early care services directed at young children with or at risk of developmental disabilities [1,2,3,4,5,6,7]. These approaches aim to promote children’s language development while supporting the family during this critical period [7]. They are based on the social interactionist perspective of language acquisition, which maintains that language is learned in the context of social interaction and is driven by the social purpose of communication [8]. In this sense, significant adults in children’s life play a critical role in interpreting their intentions and providing language that maps the child’s interests and focus [9]. Consequently, this type of intervention encourages parents to use a responsive communication style that is well adjusted to their child’s level of communication development. The contingency between a child’s utterances and the adult’s responses (e.g., imitation, expansion), facilitates language processing, allowing the child to redirect more cognitive resources for language learning. Some of the programs that follow this approach are, among others, *Milieu teaching* [10], *Responsive interaction* [11], *Responsivity Education/Prelinguistic Milieu Teaching* [12] and *It takes two to talk-The Hanen program for parents* (ITTT) [13], which will be the focus of the present study.

### 1.1. It Takes Two to Talk—The Hanen Program for Parents 

One of the main goals of ITTT [13] is to empower parents to become their child’s primary language facilitator by maximizing the child’s opportunities for communication development in everyday situations [14]. It may be delivered in groups (8 families maximum) or individually, and it is led by a Hanen-certified speech-language therapist.

The program aims to facilitate positive, reciprocal, and frequent interactions between parents and children, while training families in the use of language characterized by responsiveness, namely, adapted to the communicative development level of the child, and employing three kinds of linguistic facilitation strategies. The first category of strategies is child-oriented, for example, responding to the attentional focus of the child, following the child’s lead, adjusting to the child’s style and abilities, organizing the environment to increase the communicative initiations of the child, or being at his/her physical level. The second category of strategies promotes interaction by waiting expectantly, providing cues for the child to take his/her turn, or reducing directiveness, among others. Lastly, the third category consists of strategies that are based on modeling language, commenting on the activities carried out by the adult and the child, labeling referents in a contingent way, using simple, short, and repetitive utterances, or expanding the child’s turns. These strategies are explained, illustrated, and practiced in simulated situations during eight group sessions. Afterward, they are applied at home, so that the intervention becomes a continuous process, which takes place throughout the day. During ITTT, families receive three individual video feedback sessions from the speech-language pathologist, with the purpose of practicing the strategies in real-life contexts, reviewing the child’s goals, and discussing any issues parents may consider. After the program, a new assessment is made in order to verify changes in the children’s language development and in the parents’ communication style. A more exhaustive description of ITTT can be found in Girolametto, Weitzman, and Drake [14].

### 1.2. Research on ITTT: Children and Parents’ Outcomes

A series of studies have been conducted in order to determine the effectiveness of ITTT both in parents and their children. In the first investigation on this topic, participants were 20 families and their children aged between 22 and 62 months, diagnosed with language disorders of varied etiology [15]. After attending the program, the mothers in the intervention group changed their communicative style: they talked less, used a more balanced ratio of turns, and were able to maintain longer conversational exchanges with their children in comparison with the mothers in the control group. In addition, the children in the treatment group initiated more topics, were more responsive to their mothers’ preceding interventions, and used more verbal turns and a more diverse vocabulary than the children in the control group. With the aim of extending these findings, a subsequent randomized controlled study included a larger sample of 32 families of children with developmental and language disorders [16]. After the intervention, the mothers in the experimental group used more strategies promoting language development compared to the mothers in the control group, and this progress was maintained four months later. However, these investigations, which revealed changes in the mothers’ communication style, did not show effects in objective measures of children’s language performance. Thus, when the Hanen Program was compared to a direct clinical approach, children receiving both interventions obtained similar scores in standardized language assessments and in grammar indicators (e.g., mean length of utterance), and the expected level of statistical difference was not observed [17].

The lack of statistically significant results led to the design of a new version of the program, including the focused stimulation technique. This technique involves the modification of the input that the child receives in order to facilitate the processing, organization, and integration of linguistic information. These changes entail the repetitive and concentrated production of certain linguistic structures that the child hears while interacting with his/her parents, who may elicit or not the planned target language [18,19].

With the addition of the focused stimulation technique, the results of the investigations on ITTT revealed significant improvements in children’s language. In a pilot study involving 16 mothers of children with expressive development delay, Girolametto, Pearce, and Weitzman [20] found statistical differences between the control and treatment groups as regards the number of target words used in a language sample obtained after the program’s implementation. However, significant differences were not found for vocabulary size, assessed through the MacArthur Communication Development Inventory (MCDI) [21]. This exploratory study was extended a year later with 25 children, with an age range from 23 to 33 months, most of them experiencing significant delays in expressive vocabulary [22]. Following the intervention, significant changes were noted concerning both the mothers’ communicative behavior and the children’s linguistic development in terms of phonology, vocabulary, and grammar.

Besides analyzing its effectiveness with late talkers and children with developmental delays, Girolametto, Weitzman, and Clements-Baartman [23] applied the Hanen Program to children with Down Syndrome. As in previous studies, the mothers talked less and employed significantly more target words during free play, in comparison with the control group. However, statistically significant differences were not found in the children’s lexical development assessed through the vocabulary section of the MCDI [21].

In contrast with the studies devoted to analyzing the effectiveness of ITTT in linguistic and communicative indicators, there are very few investigations dedicated to examining the possible indirect effects on parents’ wellbeing. Communication impairments seem to play an influential role in parents’ stress, as indicated by the significant associations found between families’ stress and the inability to achieve effective communication with their children [24,25]. Moreover, parents’ physical and psychological distress may affect the frequency and quality of the interactions with their children, undermining their ability to perceive and respond to their children’s communication acts [26]. This may negatively interfere not only with the functioning of the family unit but also with the outcomes of the intervention carried out with the child.

Results relating to the effects of parent-implemented programs on family stress are not conclusive. On the one hand, as long as the family is instructed in strategies to improve communication with their children, a greater sense of parental competence and a decrease in stress level may be expected. Indeed, Tannock, Girolametto, and Siegel [16] confirmed a stress reduction in the family following the intervention with the Hanen Program ITTT. However, other studies into similar approaches involving parents of children with neurodevelopmental disorders have reported different findings, and fail to identify significant changes in parents’ stress [27], although they have highlighted the mediator role of the parents’ perceptions of the severity of their children’s communication deficits [28]. On the other hand, it seems reasonable to think that these approaches may place excessive demands on the family, who may feel overwhelmed by not being able to satisfactorily accomplish the intervention proposals, even integrating them into daily routines [29]. In fact, Brinker, Seiger, and Sameroff [30] noted higher stress levels in a group of mothers after their weekly participation in an early care program, especially in those mothers exhibiting clinical levels of stress before the intervention. These findings differ from those of Robertson and Weismer [31], who reported a significant decrease in child-related parental stress after a direct clinician-implemented intervention with late-talking toddlers.

In sum, it is important that research on parent-implemented interventions analyze the outcomes both in children’s development and in parents’ wellbeing, as they are the main intervention agents. Previous investigations into the effectiveness of ITTT are limited, including participants with very heterogeneous cognitive and linguistic levels, and most of them were conducted by the same research team, more than two decades ago and in English-speaking contexts. In addition, the effects on children’s social communication abilities have been addressed only briefly, although they are particularly pertinent from an interactional and socio-pragmatic perspective on language acquisition, which emphasizes social interaction as a fundamental pathway to acquiring language [32,33]. With respect to the results relating to parents, it is important to analyze not only their changes in communication style but also their mental health, considering the prominent role that parents take in this kind of approach, as well as the impact of stress on the intervention outcomes.

### 1.3. Objectives 

This exploratory study into the effectiveness of the Hanen Program ITTT aims to address the following objectives:

(a) To analyze the possible advances in the language development of late-talker children following the implementation of the ITTT program, specifically, in global expressive and receptive measures and indicators of social communication, comparing these results with those obtained from a group of children receiving speech-language therapy through a clinician-directed approach.
(b) To examine if there are significant changes in parents’ stress and perceptions of their children’s communication abilities, comparing families that have experienced the ITTT program with families whose children have been involved in a clinician-directed intervention.

### 1.4. Hypothesis

According to previous research, several hypotheses were raised:

(a) It is expected to find similar results in children of both groups in standardized structural measures of language development.
(b) It is expected to find higher outcomes in indicators of social communication in the group experiencing the ITTT program.
(c) It is expected to find that parental involvement in the ITTT program does not increase their stress levels.

Hypothesis *a* and *b* are based on the nature of the ITTT approach, which teaches strategies to promote the frequency and quality of socially communicative interaction as the fundamental basis of language acquisition. With respect to hypothesis *c*, and in line with previous literature, it is anticipated that the Hanen program will not intensify the parents’ stress level. However, different factors may hinder raising a hypothesis in a clear direction. On the one hand, it is likely that if the parents observe positive changes in their children’s language abilities, they may experience a decrease in stress level. Furthermore, the families who attend the program themselves may serve as a buffer against stress, since the group format can foster a feeling of mutual support [34,35]. On the other hand, it is also possible that the program characteristics may increase the degree of self-criticism and demand that the parents place on themselves, perceiving a greater burden in their roles as parents. In addition, the group session structure may inevitably lead them to make comparisons between children who are following a faster developmental trajectory over the course of the intervention, which may contribute to increased anxiety for the families.

This pilot study extends previous research by analyzing variables related to children’s social communication as well as parents’ perceptions of their children’s language development. Furthermore, the socio-cultural context in which the study is developed is different from that of former research, which is especially relevant considering cultural and familial differences as regards language teaching beliefs and communication patterns [36]. Likewise, the type of statistical procedure employed, the Bayesian analysis, represents a new approach to data treatment. The present study is carried out with small data samples under longitudinal measurement interventions. In this respect, Bayesian probability theory allows us to add value to the *p*-values, which are strongly affected by the sample size, and its interpretation by testing the null hypothesis can be unintuitive [37].

## 2. Methods

### 2.1. Study Design

This study followed a quasi-experimental methodology, comparing the results obtained from two groups of families and their children, who experienced either the ITTT program or conventional clinician-directed therapy without the ITTT component. Children and parents were assessed at two time points: the first assessment was carried out the month immediately before starting the intervention (Time 1); the second assessment took place about 6 months later, after the therapy was finished (Time 2). Two speech and language therapists carried out the children’s evaluation, and one psychologist was involved in the parents’ assessment. Even if the therapists knew the general purpose of the study, they were blind to the group status of the children and the families.

### 2.2. Participants

Families were contacted through Early Care Centers in Valencia, where they were offered the possibility of attending an informative session about the Hanen ITTT Program. Sixteen families attended this session, where the characteristics of the program, the expected schedule, the participation criteria, and all the information related to this research were explained. Specifically, the participation criteria for families and children were as follows:

- Families had to have children with communication and language delays that attended Early Care Centers in Valencia (Spain), and who were within an age range of 18 and 40 months.
- The children had to meet the usual criteria employed to identify late-talking children [38]: at 24 months, having an expressive vocabulary lower than 50 words and/or not making two-word combinations in spontaneous language; at 18 months, obtaining a percentile equal to or lower than 10 in the vocabulary section of the *MacArthur–Bates Communicative Development Inventories* (MCDI) [21,39] (see description in Section 2.3.1); not having sensory, cognitive, neurological or emotional disorders (for example, autism spectrum disorder, cerebral palsy, brain damage, etc.). Moreover, children had to obtain scores within the range of two or more standard deviations below the mean for children of the same age in the Developmental Reynell Developmental Language Scales (RDLS III) [40] (see description in Section 2.3.1).
- Children’s families had to live in the same home as the child. They had to be Spanish first-language speakers or bilingual in Spanish and Valencian (the other official language spoken in Valencia).

Of the sixteen families that were interested in attending the program, three were excluded because their children’s scores in standardized assessments indicated a language performance in the mean of their chronological age. Therefore, 13 families joined the intervention, but three of them were excluded from the investigation because, during the program, subsequent evaluations revealed that their children did not meet the established inclusion criteria. Consequently, the final sample was composed of 10 families and 10 children with ages ranging from 18 to 40 months and a mean age of 29 months (standard deviation = 7.09 months).

The control group, which was contacted following the same procedure as the treatment group, was composed of families and children with similar characteristics in terms of gender, age, language, parents’ sociocultural level, family typology, and mother tongue. The families and children in this group were attended by professional teams in Early Care Centers and did not want to enroll in the ITTT program due to time and/or availability limitations. However, they agreed to participate in the present investigation. Of the 9 families that were potentially eligible to form the control group, 2 were excluded from the study: in one case, the child did not meet the inclusionary criteria and, in the other, the family was not available for the second assessment. Therefore, the final control group was composed of 7 families and 7 children with ages ranging from 18 to 38 months and a mean age of 29 months (standard deviation= 8.7 months). Table 1 and Table 2 show the sociodemographic characteristics of all families and their children.

All the procedures carried out in this study followed the ethical norms stated in the Declaration of Helsinki in the European Council Agreement (1964). The study received full ethical approval from the Research Ethics Committee of the Universidad Católica de Valencia (Code: UCV2018-2019-031). Every participant signed an informed consent form before starting the research. In this document, they were informed about their voluntary participation in the program, the confidential treatment of the data, and the right to withdraw their consent at any time.

Table 1 depicts the demographics of families across groups involved in the current study. This information includes age, gender, marital status, type of family, family size (mean number of children), education, and occupation. It should be noted that there were no cases of families where both parents were unemployed. As can be seen, both parents of each child participated in the study, except for one single-parent family in each group, where the participant was the mother.

Table 2 depicts specific information about children’s characteristics in terms of expressive communication, receptive communication, cognition, and global development according to the results of the Battelle Developmental Inventory (BDI) [41], a standardized instrument that evaluates the fundamental abilities involved in child development. Assessment results were gathered through structured tasks, parent interviews, and observation of the child in natural environments.

Both groups of children were matched according to gender, age, cognitive level, and expressive language, using the raw scores obtained in the vocabulary section of the MCDI-Spanish adaptation [42], the Expressive Language Scale of the Reynell Developmental Language Scales (RDLS-III) [40], and the Cognitive Area of the Battelle Developmental Inventory (BDI) [41]. They were also matched according to families’ sociodemographic variables like parents’ age, language spoken at home, family typology, and socioeconomic level. For this purpose, the composition percentages were tested to reach similar groups, as depicted in Table 1. No differences were found across sex (χ^2^_(1)_ = 0.1; *p* = 0.96) and type of family (χ^2^_(1)_ = 0.07; *p* = 0.78) across groups. Moreover, no statistically significant differences were found for the children’s age (W = 103; *p* = 0.37), mother’s age (W = 47.5; *p* = 0.88), or father’s age (W = 37; *p* = 0.60) under the Mann Whitney U test.

### 2.3. Measurement Instruments

#### 2.3.1. Children’s Measurements

- *MacArthur–Bates Communicative Development Inventories- Spanish Adaptation* (MCDI-S) [42]. This is a parental report instrument and a reliable and valid tool for evaluating the expressive vocabulary and syntactic development of toddlers. It has also been used in several parent-implemented language intervention investigations involving late talkers [20,23]. There are two forms of the MCDI-S: words and gestures, for children aged 8 to 16 months, and words and sentences, for children aged 16 to 30 months. In this study, this last form was used, consisting of a list of words divided into several categories in addition to a section on grammar production that assesses first word combinations, emerging syntax, and the use of various morphological forms. In this study, sections on vocabulary and grammar (Word Endings and Morphosyntactic Complexity) were used. The Spanish adaptation of the MCDI [42] has good psychometric properties such as internal consistency. Specifically, Cronbach’s alpha values are 0.99, for vocabulary, and 0.99 for morphosyntactic complexity.
- *Reynell Developmental Language Scales (RDLS-III)* [40]. RDLS assesses the expressive and receptive language of children aged 1 to 7 years old, with a focus on vocabulary and grammar. The receptive scale is composed of 62 items, organized into 10 sections that assess comprehension, from single words to complex grammar, and inferential skills. The children have to manipulate toys or point to an image in response to the evaluator’s instruction. The expression scale contains 62 items which are organized into 6 main sections. A distinction is made between single words and syntactic sequences. Toys, images, and puppets are used to elicit the children’s answers, using a series of procedures. The RDLS-III is based on a robust standardization that allows for the identification of children with language disorders and it has a remarkable degree of reliability and validity (Cronbach’s alpha of 0.97 for the receptive scale and 0.96 for the expressive scale).
- *Communication and Symbolic Behavior Scales Developmental Profile (CSBS DP)* [43]. The CSBS DP is a standardized instrument designed to evaluate the social, communication, and symbolic abilities of children whose functional communication age is between 6 months and 2 years. In detail, it evaluates 7 language predictors: (1) Emotion and Use of Eye Gaze, (2) Use of Communication, (3) Use of Gestures, (4) Use of Sounds, (5) Use of Words, (6) Understanding of Words, and (7) Use of Objects. This instrument comprised three components: a checklist completed by the parents, a caregiver Questionnaire, and an observational measure, which was the component used in this study. It consists of a face-to-face evaluation of the child interacting with a parent and a clinician during a series of communicative situations designed to entice the child to communicate spontaneously. Materials used during the evaluation include action-based toys, books, and play materials that assess how a child uses and plays with objects symbolically and constructively. It takes approximately 20 min to administer, and it yields total scores and three composite scores: (1) social, (2) speech, and (3) symbolic. In order to assess the child’s social communication abilities, the raw scores obtained in the Social composite of the scale were used. This scale includes Emotion and Eye Gaze, Communication, and Gestures. Emotion and Eye Gaze involves measuring the child’s use of gaze shifts for social referencing during interactions, measuring sociability or sharing of positive affective states, and measuring gaze/point following. Communication includes the rate and function of communicating, that is, the number of communicative acts and the purposes they serve (behavior regulation, social interaction, and joint attention). Finally, Gestures encompass the variety of conventional gestures and the sophistication of hand gestures from contact to distal. The CSBS DP has adequate psychometric properties of reliability and validity. The Cronbachs’ alpha of the different subscales ranges between 0.73 and 0.95. The scores in the CSBS DP have proven to be a powerful predictor of receptive and expressive language outcomes at the age of 3 years and older [44].

#### 2.3.2. Parents’ Measurements

- *Parenting Stress Index-Short Form (PSI-SF)* [45,46]. This is one of the most commonly used measurement instruments of parenting stress both in clinical and research contexts. The PSI-SF is a 36-item self-report instrument including three subscales. Each subscale consists of 12 items rated from 1 (strongly disagree) to 5 (strongly agree), with subscales scores ranging from 12 to 60. The Parental Distress subscale (PD) assesses the stress that parents are experiencing in their roles as parents (e.g., “I often feel I cannot handle things well”); the Parent-Child Dysfunctional Interaction subscale (PCDI) is focused on parents’ perceptions of their expectations about their child (e.g., “My child rarely does things for me that make me feel good”), and the Difficult Child (DC) subscale is centered on the behavior of the child that facilitates or hinders his/her education (e.g., “My child seems to cry or fuss more often than most children”). A total score is calculated by adding together the three subscales scores, ranging from 36 to 180. Scores of 90 or above may indicate a clinical level of stress. Abidin [43] reported Cronbach’s alpha coefficients of 0.91 for PSI-SF total score and 0.87, 0.80, and 0.85 for PD, PCDI, and DC subscales respectively. The Spanish adaptation has also revealed good psychometric properties [46].
- *Parent Perception of Language Development (PPOLD)* [28,47]. The PPOLD is a questionnaire composed of 20 items, grouped into two factors: *success*, which measures parents’ perceptions relating to how well they are affecting their child’s communication development (e.g., “My efforts working on communication with my child seem to be paying off”); and *difficulty*, which measures parents’ perceptions relating to the severity of their child’s communication problems (e.g., “Helping my child learn to communicate is more work than I thought it would be”). This instrument, therefore provides an indication of parental stress in relation to the child’s communication abilities. Each item is rated on a Likert scale from 1 (strongly disagree) to 5 (strongly agree). The questionnaire has good internal consistency, with alphas above 0.70 for both factors (success and difficulty). Both instruments, the PSI-SF and the PPOLD were filled out by the caregiver who was most involved in the intervention process. In the case of the ITTT program, the parent who consistently attended the sessions, and was therefore most engaged in the intervention, completed the questionnaires.

### 2.4. Intervention Description

#### 2.4.1. ITTT Hanen Intervention

The ITTT program was delivered in a group format and was led by two speech and language pathologists, trained and certified by the Hanen Centre. The 10 final participating families were randomly split into two groups of 5 families, each group being led by one therapist. Before the program implementation, an assessment of the child’s linguistic profile, the family communication style, and their interaction pattern, was carried out, and communication goals were set to be reached along the course of the intervention. A typical group session lasted approximately 2 and a half hours and included a combination of interactive presentations, group discussions, video analyses, and opportunities for practicing the strategies taught through role-playing. 

During these eight sessions, which were held on Saturday mornings in a classroom of the Universidad Católica de Valencia, parents followed the manual of ITTT and completed action plans specifying the strategies to use in order to achieve their children’s communication and linguistic goals. The content of the first five sessions focused on the description of the stages of communication development and the introduction of different strategies addressed to promote language learning: waiting for the child to initiate an interaction and responding sensitively; following the child’s lead; matching parents’ turns to their child’s turn, or asking questions to encourage conversation. In addition, the families learned to model language at their child’s level by labeling what the child is focused on and expanding the child’s words (i.e., by adding a syntactic or semantic element). 

Parents also were shown how to stimulate their child’s receptive vocabulary by using more decontextualized language when commenting on the child’s activity or topic, and the ways to choose specific communication goals and contexts for using them. The last three sessions were devoted to applying the strategies to communicative contexts around books, play, and music. After the second, fourth, and seventh sessions, three home visits were scheduled with each family. These individual visits consisted of videotaping a brief segment of the parent-child interaction, which was subsequently reviewed and discussed with the speech-language therapist, who provided immediate feedback. There were no group sessions during the weeks when video feedback sessions were carried out.

#### 2.4.2. Clinician-Directed Therapy

Clinician-directed therapy was characterized by following a one-to-one traditional behaviorist approach where the therapist interacts directly with the child to elicit a response. The clinician specifies materials to be used, how they should be utilized, the type and frequency of reinforcement, the form of the responses to be accepted as correct, and the order of activities [48,49]. Given that late-talkers have a small vocabulary, these activities were intended to increase the lexical repertoire and, when possible, to work on syntax (e.g., promoting two-word combinations). The most common educative procedures employed by the therapists were modeling and imitation. This approach is less naturalistic than ITTT since it involves total control on the part of the professional and is developed in the clinical context. The therapist may demonstrate to the parents the different techniques that promote language, encouraging them to adopt these strategies in their interaction with their children outside therapy sessions (e.g., “try this at home” [50]). However, the focus is on clinician-child curricula rather than parental implementation [51], and learning occurs mostly within the treatment activities. In other words, as opposed to the Hanen approach, the adult learning perspective is not considered, since the speech-therapist does not consistently explain to the parent what he/she is doing and why. Therefore, the main difference between both kinds of intervention is that, in the clinician-directed approach, the center of attention is the child’s language, the therapy is carried out in the structured setting of the speech therapy room, and the professional is the main agent of the intervention. In contrast, ITTT adopts a family-centered perspective where the focus is much more on the parents’ language and interaction style [17]. Regarding the timing of the intervention, children in this group received between one and two weekly sessions over approximately four months, and these were delivered by different speech and language therapists working in Early Care Centers and Speech Therapy Clinics in Valencia, who had not been trained in the ITTT program.

### 2.5. Data Analysis 

After the descriptive analyses, a non-parametric approach was chosen. It should be noted that sample size calculation is a common concern in many fields, as an adequate sample size minimizes the risk of Type II error or not detecting a statistically significant difference where one exists. Given the characteristics of the current sample due to a longitudinal approach inherent to the intervention, the adequate sample size was smaller than other studies that do not use this design. For this reason, Bayesian inference was also carried out. Adopting this approach brings several advantages over frequentist null-hypothesis significance testing, such as providing evidence in favor of the null hypothesis and discrimination [52], accounting for prior knowledge [53], and removing the need to pre-specify sample size [54]. For this reason, a Bayesian Mann-Whitney test was chosen. The scores were transformed into amplitude (Δ) between pre and post moments. Data analysis was performed using JASP (Version 0.12.2, University of Amsterdam, Amsterdam, the Netherlands) (Computer software, https://jasp-stats.org/faq/how-do-i-cite-jasp/ (accessed on 30 July 2021)).

## 3. Results

Table 3 depicts the descriptive analysis for the variables related to children’s language and social communication. After performing the Mann-Whitney nonparametric U test across groups, no statistically significant differences were found (*p* > 0.05). On the other hand, the descriptive analysis of the variables related to parents’ stress and perceptions of their children’s communication difficulties are described in Table 4. Once again, no statistically significant differences were found (*p* > 0.05) under the Mann-Whitney nonparametric U test across groups.

An alternative strategy was chosen. In this way, a Bayesian Mann-Whitney test was carried out on the distances of the pre- and post-moment scores, henceforth described as Δ, as depicted in Table 5.

The Bayes factor notation (BF_10_) was employed to examine which hypothesis is more probable. Therefore, BF_10_ is used as a measure of evidence to support the H_1_ (the alternative) over H_0_ (the null hypothesis). A large BF_10_, therefore, indicates a change in beliefs towards H_1,_ being CSBS SC. Scale total the larger one.

**Figure 1:**
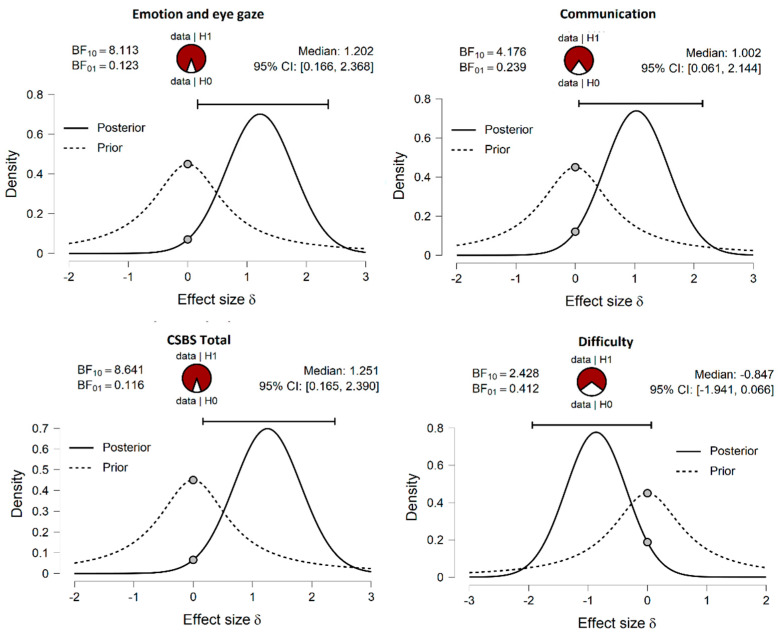
Bayesian inferential plots on prior knowledge and posterior knowledge, to indicate evidence to support the H1 over H0 under the Bayes factor notation (BF_10_).

With regard to Figure 1, the pizza plot distribution shows the proportion of evidence for the H_1_ (red) and H_0_ (white) hypothesis, being higher for these four cases but not for the other variables. Moreover, the dashed line shows the prior distribution, while the solid line the posterior distribution based on the current data. As described in previous literature [55], the posterior distribution is shifted to the right over positive effect sizes, as depicted for Emotion and Eye Gaze, as well as Communication. The other way around occurs for Difficulty. It should be noted that each distribution has a grey dot at the 0.0 effect size (δ), showing whether the dot on the prior distribution is higher than the one on the posterior distribution. Therefore, the Bayes factor supports the alternative hypothesis in this direction.

## 4. Discussion

The present study aimed to update knowledge of the effectiveness of the Hanen Program ITTT in Spain and focused on two objectives. The first objective was to analyze changes in late talkers’ language and social communication after the implementation of ITTT, comparing these outcomes with those obtained from a group of children receiving speech-language therapy through a clinician-directed approach. Both groups progressed in language development in similar ways, in some cases even getting close to the mean of their group of reference. However, as predicted, no significant differences were found between them in standardized measures of expressive and receptive language, as well as in parents’ estimates of expressive vocabulary, assessed through the MCDI-S [42]. These results echo the findings of previous investigations into the Hanen approach that have used other kinds of standardized tests [16] or the same parental reports of language development [17,20]. It is possible that standardized measures, which assess global language performance in general terms, may not capture the subtle changes in language acquisition within such a limited time period. Therefore, it would be desirable to make use of more fine-grained measures such as micro-analytic techniques of language sample analysis, in order to perceive these changes, as shown in previous investigations with ITTT and/or the focused stimulation technique [18,19,20].

In contrast, instruments used to assess social pragmatic aspects of communication, specifically, emotion and gaze and communication, allowed us to identify significant differences between groups. These findings coincide with results reported in other studies using the Hanen approach, with an increase in the children’s turn-taking abilities [15, 22}, along with longer communication exchanges [16].

The outcomes in terms of social communication are expected due to the nature of the strategies taught throughout the program, most of them focusing on the interactive nature of communication, aimed at initiating the interaction (staying at the child’s level, face to face), and keeping the interaction going (adjusting the turns to the child’s turns, cueing the child to take the turn and repeating this kind of interaction in daily routines). Although ITTT includes strategies to enhance vocabulary development (through focused stimulation, among other strategies), the majority of them stress the interactional features of high-quality input [56], and it is precisely the interactive dimension of the children’s communication development, social communication, that shows the greatest advances in the present study. In this sense, Del Rio [57] pointed out that naturalistic interventions may not be equally applied to all language components. The current study’s outcomes revealed that a parent-implemented intervention such as ITTT achieves significantly more effects in the pragmatic component of language in comparison with a clinician-directed approach. This is a relevant finding, considering the predictive relationship between social communication behaviors (acts for joint attention, for example) and later expressive language in late-talking toddlers [58]. However, more formal aspects of language, such as vocabulary and grammar, seem to benefit equally from both intervention modalities.

With respect to the second objective, the effects of ITTT on parents’ stress, although the scores obtained in the PSI SF [45,46] were lower for the Hanen group, which indicates lower stress, the results did not evidence significant differences either between both groups or intragroup. These findings align with those of other investigations [27] and allow us to suggest that the Hanen approach does not increase parents’ stress levels. It is important to underscore that in our study, the stress level did not reach the clinical threshold in either group before the intervention. However, other investigations have reported that mothers of late talkers rate their relationship with their child as significantly more stressful on the Parent-Child Dysfunction scale of the PSI SF [59] and this factor has to be taken into account when designing interventions for late-talking children.

In addition, parents’ perception of their children’s communication development was significantly better in the Hanen group in comparison with the clinician-directed modality. Concretely, the PPOLD [28,47] revealed that they perceived a shorter gap between their children’s language level and the typical linguistic development. In contrast, the families in the direct intervention group did not express these perceptions and had more difficulties finding the time to help their children learn to communicate, highlighting the stress that language therapy implied. The different pattern of results shown by the Hanen group might be due to the characteristics of the ITTT approach, which teaches parents a responsive communicative style that becomes fluent and automatic, and families learn to smoothly integrate language learning opportunities into everyday routines. Therefore, it is not a matter of finding time to stimulate children’s language but of adopting a communicative style in everyday situations in natural environments.

These contrasting outcomes in the Hanen group, which show that parents’ positive perceptions about their children’s language development increased, coincide with previous data on social validity in the Spanish context, and the acceptability and feasibility of the procedures used in the ITTT program [60]. Nevertheless, the lack of significant changes in general stress level measurements leads us to reflect on the need to complement this type of program with specific treatments when parents’ stress exceeds the level of clinical significance, which may impact the interaction patterns and, consequently, the success of the intervention carried out with the children [26].

Late talkers are of great interest to early service providers and families, considering that early language deficits are significant risk factors for many disorders [2]. Along with the advantages in the area of social communication found in the present study, and the improvement in parents’ perceptions of the difficulties of their children, other factors must be taken into account when selecting interventions aimed at this population. One of them is cost-effectiveness, an issue already addressed by Gibbard, Coglan, and MacDonald [61]. In this study, in comparison with the 8 sessions (and 3 individual visits) of ITTT, the clinician-directed therapy involved a substantially greater amount of sessions (more than double in the four months of intervention), which implies greater financial costs for the families and more time investment for the speech-language pathologists, who usually have heavy caseloads. In another vein, recent research has revealed the effects of the program on children’s overall social-emotional functioning [62], which strengthens the empirical support for this approach.

## 5. Limitations

This study presents some limitations that need to be acknowledged. First, the sample size is very small, although this can be considered a sufficient number when reviewing the meta-analysis carried out by Roberts and Kaiser [6], where the minimum number of participants of the investigations included was 12 members. Second, the participating families came from middle socioeconomic and sociocultural backgrounds, which is a common shortcoming of research on parent-implemented language interventions [1,2]. Furthermore, the motivation to participate in the program is a factor that should be considered as it might have had a moderator role in the results. Other concerns relate to the imbalance of the number of participants in the treatment and control groups and the lack of randomized allocation. In addition, information about the time being involved in previous interventions was not collected, and this is an aspect that might also have an influential role in the outcomes. Likewise, in the case of ITTT, treatment fidelity was examined using parental attendance data and observation of parent-child interaction during the three home visits, a method previously used in other studies with the Hanen Program [20]. However, it would have been desirable to employ a more exhaustive and systematic measurement of implementation fidelity.

Regarding the measures assessing children’s social communication abilities, although the instrument used has the advantage of being a standardized test based on a validated scoring, it would have been convenient to obtain these data using the information provided by the family, which is more ecologically valid, and particularly relevant when assessing pragmatics. Finally, the results were only obtained at the end of the intervention and it would have been interesting to collect data some months later, in order to see whether changes are maintained or whether new significant differences are found.

## 6. Conclusions

This exploratory study makes a small contribution to the available data about parent-implemented program interventions aimed at late-talking children. When compared to a clinician-directed therapy, the effects of the Hanen Program ITTT were similar on linguistic variables such as vocabulary or syntax complexity, but significantly greater on children’s social communication. This is an important finding because extant research has stressed the predictive value of social communication abilities on subsequent expressive and receptive language performance in late-talking toddlers [58].

Furthermore, parents who took part in the ITTT program changed their perceptions about their children’s language difficulties in a positive way. This is also a relevant finding, considering the studies that demonstrate the mediator role of these perceptions between the child’s expressive language and parental stress [28]. In this respect, none of the intervention modalities either increased or decreased families’ stress, which is in line with the results of previous investigations [21]. These outcomes suggest that parent-implemented programs do not overwhelm families, but they also point to the need to be aware of the implementation of specialized interventions in situations of clinically significant stress.

To sum up, parents of late talkers must make informed decisions about scientific-based effective interventions. But this is only one aspect of evidence-based practice which must also take into account clinical expertise and the client’s preferences and values. The confluence of these three factors will ultimately lead families to choose one or other of these intervention modalities.

## Figures and Tables

**Table 1:** Sociodemographic characteristics of the families.

	Treatment Group Hanen ITTT-Families (*n* = 10)			Control Group Clinician Directed-Families (*n* = 7)	
	Adults	*n* = 19	%	*n* = 13	%
**Gender**	Men	9	47.3	6	46.1
	Women	10	52.6	7	53.8
**Type of family**	Single-parent	1	10	1	14.2
	Two-parent	8	90	6	85.7
**Family size** **(mean number of children)**	One	3	30	3	42.8
	Two	7	70	3	42.8
	Three	0	0	1	14.2
**Language**	Spanish	9	90	6	85.7
	Bilingual	1	10	1	14.28
**Paternal education**	Secondary	6	40	2	33.3
	Post-secondary	4	60	4	66.6
**Maternal education**	Secondary	4	40	3	42.85
	Post-secondary	6	60	4	57.14
		**Mean**	**Range**	**Mean**	**Range**
**Mother’s age**		34.9	32–42	36.5	32–41
**Father’s age**		35.6	32–39	34.9	32–47
**Fathers employed**		9	100	4	66.6
**Mothers employed**		7	70	5	71.46

**Table 2:** Children’s characteristics in terms of communication, cognition, and global development.

	Treatment Group Hanen ITTT Children (*n* = 10)		Control Group Clinician Directed (*n* = 7)	
**Gender**	Male: 5		Male: 4	
	Female: 5		Female: 3	
**Age**	Mean	Range	Mean	Range
	29.6 months	18–40	29.8 months	18–38
	Mean	SD	Mean	SD
**BDI Exp.**	5.9	2.42	5.4	2.14
**BDI Rec.**	6.70	3.20	8.57	3.10
**BDI Cogn.**	14.00	2.94	16.14	5.75
**BDI Total**	12.80	5.43	14	5.16

SD = Standard Deviation. BDI: Battelle Developmental Inventory. Exp.: Expressive Communication. Rec.: Receptive Communication. Cogn.: Cognitive.

**Table 3:** Descriptive statistics related to children and Mann-Whitney nonparametric U test across groups.

		Control (*n* = 7)		Hanen (*n* = 10)			
		Mean	*SD*	Mean	*SD*	*W*	*p*
Pre	CSBS DP Emot.	28.714	9.928	29.700	7.804	36	0.96
	CSBS DP Com.	29.571	9.378	22.000	6.325	15	0.05
	CSBS DP Gest.	13.429	5.533	14.600	4.427	39	0.73
	SC Scale Total		22.50	66.30	16.06	24	0.31
Post	CSBS DP Emot.	30.429	10.596	38.500	6.151	53	0.08
	CSBS DP Com.	32.714	9.621	32.800	6.143	32	0.80
	CSBS DP Gest.	17.857	4.220	21.200	1.874	53.5	0.05
	SC Scale Total	81	21.87	92.50	12.36	46	0.31
	RDLS-III Comp.	20.42	14.22	12.70	11.49	22	0.22
Pre	RDLS-III Expres.	5.85	5.66	7.90	8.31	39.5	0.69
	RDLS-III Total	13.13	9.94	10.3	9.9	79.50	0.31
Post	RDLS-III Comp.	30.57	15.54	27.40	10.85	27	0.46
	RDLS-III Expres.	12	8.86	12.30	3.91	38.50	0.76
	RDLS-III Total	21.28	12.2	19.85	7.38	85.50	0.67
Pre	MCDI Vocab.	109	145.86	129.90	161.94	41	0.60
	MCDI W End.	4	5	3.90	3.47	38.50	0.76
	MCDI M Compl.	12.70	13.02	12.70	13.02	37	0.88
Post	MCDI Vocab.	343.42	162.52	330.70	154.50	34	0.96
	MCDI W End.	11.57	3.77	8.80	2.48	19.5	0.13
	MCDI M Compl.	29.28	8.74	29.50	5.10	29.5	0.61

CSBS DP. Communication and Symbolic Behavior Scales Developmental Profile. Emot.: Emotion. Com.: Communication. Gest.: Gestures. SC: Social Communication. RDLS-III: Reynell Developmental Language Scales. Comp.: Comprehension. Expres.: Expression. MCDI: MacArthur–Bates Communicative Development Inventories. Vocab.: Vocabulary. W End.: Words Endings. M Complex.: Morphosyntactic Complexity.

**Table 4:** Descriptive statistics relating to parents and Mann-Whitney nonparametric U test across groups.

		Control (*n* = 7)		Hanen (*n* = 10)			
		Mean	*SD*	Mean	*SD*	*W*	*p*
Pre	PPOLD Succ.	37.714	5.345	38.000	3.621	38	0.80
	PPOLD Diff.	13.714	5.992	14.300	3.057	37.5	0.84
	PPOLD Total	25.71	2.59	26.15	2.23	35	0.90
Post	PPOLD Succ.	39.571	2.440	40.400	2.875	37.5	0.84
	PPOLD Diff.	14.429	6.373	11.200	1.814	26	0.40
	PPOLD Total	27	2.55	25.80	1.73	23.50	0.28
Pre	PSI SF Distress	31.000	11.590	26.100	6.045	26.5	0.43
	PSI SF Dysf.Int.	21.286	7.342	21.400	3.438	40.5	0.62
	PSI SF Diff. Ch.	22.857	9.634	26.000	7.972	41	0.59
	PSI SF Total	75.14	25.77	73.50	13.09	38	0.80
Post	PSI SF Distress	28.000	10.424	25.400	9.192	25.5	0.37
	PSI SF Dysf.Int.	19.000	6.164	19.800	3.736	42	0.52
	PSI SF Diff. Ch.	21.000	5.774	21.700	5.122	37	0.88
	PSI SF Total	67.43	19.81	66.90	12.02	40	0.65

PPOLD: Parent Perception of Language Development. Succ.: Success; Diff.: Difficulty. PSI SF. Parent Stress Index Short Form. Dysf. Int.: Dysfunctional Interaction. Diff. Ch.: Difficult Child.

**Table 5:** Descriptive statistics and the Bayesian Mann-Whitney test was carried out on the Δ score.

	BF_10_	W
CSBS DP Emot. Δ	8.113	68.500
CSBS DP Com. Δ	4.176	64.500
CSBS DP Gestures Δ	0.816	50.000
CSBS SC Scale Total Δ	8.641	69.000
MCDI Vocab. Δ	0.466	32.000
MCDI W End. Δ	1.064	17.000
MCDI M Compl. Δ	0.439	35.500
RDLS-III Comp. Δ	0.682	49.500
RDLS-III Expres. Δ	0.505	28.000
PSI SF Distress Δ	0.509	43.000
PSI SF Dysf.Int. Δ	0.454	35.000
PSI SF Diff. Ch. Δ	0.483	26.500
PSI SF Total Δ	0.435	36.000
PPOLD Succ. Δ	0.451	40.500
PPOLD Diff. Δ	2.428	10.000

CSBS DP. Communication and Symbolic Behavior Scales Developmental Profile. Emot: Emotion. Com.: Communication. SC: Social Communication. MCDI: MacArthur–Bates Communicative Development Inventories. Vocab.: Vocabulary. W End.: Words Endings. M Compl.: Morphosyntactic Complexity. RDLS-III: Reynell Developmental Language Scales. Comp.: Comprehension. Expres.: Expression. PSI SF. Parent Stress Index Short Form. Dysf Int.: Dysfunctional Interaction. PPOLD: Parent Perception of Language Development. Succ.: Success. Diff.: Difficult.

## Data Availability

You can find a further description of the data in Senent-Capuz, N (2017) (doctoral thesis).
